# AMMI and GGE biplot analyses of Bambara groundnut [*Vigna subterranea* (L.) Verdc.] for agronomic performances under three environmental conditions

**DOI:** 10.3389/fpls.2022.997429

**Published:** 2023-01-20

**Authors:** Vincent Ishola Esan, Grace Oluwasikemi Oke, Timothy Oyebamiji Ogunbode, Idowu Arinola Obisesan

**Affiliations:** ^1^Environmental Management and Crop Production Unit, B. Agriculture Program, College of Agriculture, Bowen University, Iwo, Nigeria; ^2^Pure and Applied Biology Program, College of Agriculture Bowen University, Iwo, Nigeria

**Keywords:** Bambara groundnut, food security, genotype × environment interaction, AMMI, GGE biplot, multi-environment trial, stability analysis, yield

## Abstract

**Introduction:**

The two most common styles to analyze genotype-by-environment interaction (GEI) and estimate genotypes are additive main effects and multiplicative interaction (AMMI) and genotype + genotype × environment (GGE) biplot. Therefore, the aim of this study was to find the winning genotype(s) under three locations, as well as to investigate the nature and extent of GEI effects on Bambara groundnut production.

**Methods:**

The experiment was carried out in the fields of three environments with 15 Bambara groundnut accessions using the randomized complete block design (RCBD) with three replications each in Ibadan, Osun, and Odeda. Yield per plant, fresh seed weight, total number of pods per plant, hundred seed weight, length of seeds, and width of seeds were estimated

**Results:**

According to the combined analysis of variance over environments, genotypes and GEI both had a significant (p < 0.001) impact on Bambara groundnut (BGN) yield. This result revealed that BGN accessions performed differently in the three locations. A two-dimensional GGE biplot was generated using the first two principal component analyses for the pattern of the interaction components with the genotype and GEI. The first two principal component analyses (PCAs) for yield per plant accounted for 59.9% in PCA1 and 40.1% in PCA2. The genotypes that performed best in each environment based on the “which-won-where” polygon were G8, G3, G2, G11, G6, and G4. They were also the vertex genotypes for each environment. Based on the ranking of genotypes, the ideal genotypes were G2 and G6 for YPP, G1 and G5 for FPW, G15 and G13 for TNPP, G3 and GG7 for HSW, G7 and G12 for LOS, and G10 and G7 for WOS. G8 was recorded as the top most-yielding genotype. G8, G4, G7, and G13 were high yielding and the most stable across the environments; G11, G14, and G9 were unstable, but they yielded above-average performance; G14, G12, G15, and G1 were unstable and yielded poorly, as their performances were below average. Bowen was the most discriminating and representative environment and is classified as the superior environment.

**Discussion:**

Based on the performance of accessions in each region, we recommend TVSU 455 (G8) and TVSU 458 (G3) in Bowen, TVSU 455 (G8) and TVSU 939 (G6) and TVSU 454 (G1) in Ibadan, and TVSU 158 (G2) and TVSU 2096 (G10) in Odeda. The variety that performed best in the three environments was TVSU 455 (G8). They could also be used as parental lines in breeding programs.

## 1 Introduction

Bambara groundnut (*Vigna subterranea* (L.) Verdc.) is among the neglected and underutilized crops in Africa, but it has high nutritive value, thrives in poor soils, is tolerant to drought and salt stress, and is capable of producing yields in conditions where peanuts and groundnut fully fail (Temegne, 2018). Nevertheless, it remains regrettably less produced and unsatisfactorily known in tropical Africa. In many African countries, Bambara groundnut is used in many traditional festivals as part of gastronomic, social, and cultural entertainment (Massawe et al., 2005). Their leafy stalks and seeds are also used as animal feed (Brink et al., 2006). Bambara groundnut seeds are rich in carbohydrates (63%), proteins (19%), and fats (6.5%) and also contain calcium, potassium, iron, and nitrogen, making the seeds a complete food (Bamishaiye et al., 2011; De Kock, 2013). It is also composed of essential amino acids, which are vital for food security (Yao et al., 2015). It is also considered a medicinal plant, which is used to prevent many ailments such as colon cancer, nausea in pregnant women, epilepsy, diarrhea, and ulcers (Brink et al., 2006). The gross energy value of the seed of Bambara groundnut is greater than that of other legumes like lentils, cowpea, and pigeon pea (FAO, 1982; Amarteifio et al., 2002; Lacroix et al., 2003). It has been observed and proven that Bambara groundnut is of great nutritive value and produces significant yields under poor soils, yet it is still one of the world’s most neglected crops (Khan et al., 2021). It is considered a landrace for agroecological zones where it has been cultivated for years, which has been without any serious selection from farmers, though the farmers could have performed some mass selection (Zeven, 1998).

For a crop to be stable and well-adapted, it must be able to thrive in a given environment. Owing to the discrepancies in the environmental settings, crops are influenced by genotype × environment interactions (GEIs). Most of the time, fungi, viruses, nematodes, bacteria, rainfall, temperatures, soil chemistry, soil humidity, and disparities in soil type are the major environmental factors causing GEI effects (Oladosu et al., 2016), and they are also related to the genetic makeup of the genotypes. This is why genotype-by-environment interaction is of capital importance (Karimizadeh et al., 2013). Generally, crop improvement scientists are more interested in using agronomic traits such as yield and yield components with regard to GEI to detect lasting results to problems governing plant growth and development. Thus, many statistical tools and models have been put in place to analyze GEI effects under mega-environment experiments (Eberhart & Russell, 1966). GGE and additive main effects and multiplicative interaction (AMMI) model biplots associated with their components are the main models in GEI analysis (Alizadeh et al., 2017). Principal component analysis (PCA) is used to generate biplots, which allows us to understand the relationship between genotypes, environments, and GEI so as to ascertain stable and high-yielding genotypes to specific environments or across environments (Oladosu et al., 2017). Many researchers (Kaya et al, 2006; Karimizadeh et al., 2013; Alake et al., 2015; Alizadeh et al., 2017; Khan et al., 2021) demonstrated the usefulness of the AMMI and GGE methods in their study to detect potential yielding genotypes associated with stable performance across various environmental conditions. The main difference between the AMMI and GGE biplot methods is that the GGE biplot incorporates a “which-won-where” pattern, environment ranking, mean *vs.* stability, discriminativeness and representativeness of the environments, genotype rankings, and the use of singular value decomposition (SVD). Nevertheless, the two approaches complement each other and allow us to comprehend the GEI effects, the best genotypes, and the suitable environments for a better yield of genotypes. Thus, understanding the effects of GE interactions is paramount in determining the adaptation and stability of genotypes (Luo et al, 2015). To date, AMMI and GGE models have helped to sort out the complex GEI and identify potential and stable genotypes in multi-dimensional environments and to make available many breeding lines through genotype-by-environment interaction studies (Luo et al, 2015). This current study aims to identify superior genotypes with stable yield performance over a wide range of environments by evaluating the efficacy of various stability analysis methodologies. Another objective of this study was to examine how GEI influenced the agronomic traits of Bambara groundnut [*V. subterranea* L. (Verdc.)] genotypes as well as to identify the high-yielding stable genotypes to use as commercial varieties and for future breeding in various environments.

## 2 Materials and methods

### 2.1 Experimental sites

The multiple location trials were carried out at Bowen University Teaching and Research Farm Iwo, Osun State, Nigeria (7°38′N, 4°11′E) with an altitude of 322 m above sea level; at Ologuneru Ibadan, Oyo State, Nigeria (7°44′N, 3°83′E) with an altitude of 275 m above sea level; and Odeda, Ogun State, Nigeria (7°23′N, 3°53′E) with an altitude of 162 m above sea level. The seeds were planted in the open fields of the three above-mentioned environments as multi-environment trials in the 2021 growing season in Nigeria. The recorded average climatic conditions are presented in **Table 1**.

**Table 1:** Average climatic conditions in the three locations during the experiments.

Locations	Parameters	Aug	Sept	Oct	Nov	Dec
Osun (Bowen)	Min temperature (°C) Average temperature Max temperature (°C) Average humidity (%) Average rainfall (mm)	23.3	23.4	17	23.6	25.6
		25.2	25.7	25.9	27.7	27.7
		26.9	27.5	27.7	29.9	29.5
		80	81	86	76	65
		200.4	224.6	164.1	44.4	11.7
Oyo (Ibadan)	Min temperature (°C) Average temperature Max temperature (°C) Average humidity (%) Average rainfall (mm)	22.8	22.2	22.2	24.8	27.5
		25.8	26.4	27.0	28.6	29.5
		28.4	28.0	29.2	30.7	30.9
		89	86	86	81	62
		226.5	235.4	169.3	39.8	7.3
Ogun (Odeda)	Min temperature (°C) Average temperature Max temperature (°C) Average humidity (%) Average rainfall (mm)	24.3	23.9	24.4	23.3	25.6
		25.7	26.1	26.9	27.5	28.1
		27.0	27.2	28.3	29.4	30.0
		89	88	87	86	74
		242.1	254.2	139.2	32.9	4.45

### 2.2 Plant materials

Fifteen Bambara groundnut genotypes obtained from the Genetic Resources Center, IITA, Ibadan, Nigeria, are presented in **Table 2**. Five plants in the middle served as experimental units for data collection among the accessions used.

**Table 2:** The accessions of Bambara Groundnut and their collection sites.

S/N	Accessions	Origin
1	TVSU 454	Cameroon
2	TVSU 158	Ghana
3	TVSU 438	Cameroon
4	TVSU 633	Nigeria
5	TVSU 1520	Unknown
6	TVSU 939	Zambia
7	TVSU 513	Cameroon
8	TVSU 455	Cameroon
9	TVSU 643	Nigeria
10	TVSU 2096	Unknown
11	TVSU 194	Benin
12	TVSU 1611	Unknown
13	TVSU 1920	Cameroon
14	TVSU 1531	Unknown
15	TVSU 1392	Unknown

### 2.3 Field experimental design

The three multiple environment trials were set up using a randomized complete block design (RCBD) with three replications. In each replication, there were 15 plots/beds with each bed measuring 3 m × 0.5 m. The furrow spacing between each bed was 30 cm, and the intra-spacing distance between plants was 30 cm, while the interspacing distance between plants was 50 cm. The replications were separated from each other by a distance of 1 m. The total size of the experiment plot was 13 m × 12 m, leaving 1 m of spacing before the first replication and 1 m spacing after the third replication, with 15 beds per replication, and a total of 45 beds across all locations. Each replication had 11 plants per plot.

During the growing season, the recommended intercultural practices were carried out, and they include the following:

* Choice of the site: the sites picked for the cultivation of Bambara groundnut were leveled to avoid a runoff. Also, well-drained sites were picked across all locations to avoid water logging and pod rotting. Stony areas were also avoided to prevent pod damage.

* Cropping history: cowpea was planted in Bowen plots before Bambara groundnut was planted. In Ibadan plots, plantain and banana were cultivated before Bambara groundnut was planted. In Odeda plots, vegetables were planted.

* Land preparation: the lands used were cleared of all existing vegetation, plowed, and harrowed twice in order to obtain leveled seed beds. Manual or traditional means by the use of a hoe were used in soil preparation and plot making. Tillage was also employed during the land preparation process. The land clearing and preparation were carried out for 2–3 days in all environments.

* Seed sowing: the seeds were sown by hand to a depth of 2–3 cm. One seed per accession was sown on each plot in the replication. The sown Bambara groundnut seeds were not intercropped with any other crop during the planting season; i.e., sole cropping was carried out. The time of sowing across the three locations varied. In Bowen University teaching and research farms, sowing was carried out on 13 August 2021; in Ologuneru, Ibadan, sowing was carried out on 10 September 2021; and in Odeda Ogun, sowing was carried out on 16 September 2021. During sowing, the soil was dug up using the index finger, and one seed was placed into the hole and then covered.

* Weeding: weeding was carried out twice during the growing season across all locations. Manual weeding was carried out by the use of hoes and hand trowels and by hand removal or uprooting. The first weeding in Osun, Oyo, and Ogun states was carried out after 30 days of sowing, while subsequent weeding was carried out on 12–13 October 2021, 10–11 November 2021, and 17–18 November 2021, respectively.

* Irrigation and fertilizer application: during the planting season, no irrigation and no fertilizer were administered. Farming depended solely and extensively on natural rainfall.

### 2.4 Soil sampling and analysis

Soil at 0–15-cm depth was randomly sampled before the commencement of the three experiments. The standard procedure for soil analysis was duly followed, and the chemical compositions were analyzed.

### 2.5 Data collection

At harvest, yield and yield components were collected. They were as follows: total number of pods; FPS, final plant stand; FSW, fresh seed weight (g); NSPP, number of seeds per pod; YPP, yield per plant (g); HSW, hundred seed weight (g); YPPL, yield per plot (g); DSW, dry seed weight (g); FPW, fresh pod weight (g); MPN, mature pod number per plant; LOP, length of pods (mm); WOP, width of pods (mm); LOS, length of seeds (mm); WOS, width of seeds (mm); SP, shelling percentage (%); HI, harvest index; and YPPU, yield per plot of unshelled seeds.

### 2.6 Statistical analysis

All statistical analyses were performed in R statistical software version 4.1.1. Multiple comparisons of agronomic characters were used with the help of the “Agricolae” package. Combined ANOVA was carried out to test the presence of GEI. Then, AMMI and GGE biplot methods were used to analyze multivariate stability and GEI. The AMMI and GGE biplots were computed using multi-environment trial analysis (Olivoto and Lúcio, 2020). Their methods are modeled on the AMMI and GGE concepts of Yan and Kang, 2003, Yan and Manjit, 2003 and Yan et al., 2007. The GGE biplots and AMMI methods based on mega-environment assessment were used to plot the graphs of the following models: AMMI 1 and AMMI 2, which-won-where pattern of GGE, ranking of genotypes, mean performance *vs.* stability, discriminativeness and representativeness, ranking environments, and relationship among test environments. They were used to visualize the presence of G × E interaction.

## 3 Results

### 3.1 Soil analysis

Bowen soil plot was characterized by the highest percentage of sand, Bray P, OC, Mn, Na, and Mg, while the Ibadan soil plot was higher in clay and Cu; then, in Odeda, a higher percentage of silt N, Zn, Fe, K, and Ca was observed (**Table 3**). A slightly different pH was recorded: 7.20, 7.64, and 6.79 at Bowen, Ibadan, and Odeda, respectively.

**Table 3:** Physio-chemical composition of soil from Bowen, Ibadan, and Odeda.

Properties	Bowen	Ibadan	Odeda
Sand %	71.00	60.22	56.22
Clay %	10.90	29.14	13.63
Silt %	18.10	10.64	30.15
% N	0.19	0.20	1.12
Bray P	23.11	14.84	17.26
% OC	1.19	0.99	0.84
Zn (ppm)	1.38	1.30	2.30
Cu (ppm)	0.68	1.20	0.98
Fe (ppm)	77.96	82.40	89.62
Mn (ppm)	169.42	108.17	117.64
Na (cmol/kg)	0.09	0.03	0.08
K (cmol/kg)	0.43	0.27	1.23
Mg (cmol/kg)	2.50	2.00	0.75
Ca (cmol/kg)	4.75	2.78	5.13
pH	7.20	7.64	6.79

### 3.2 Combined analysis of variance for agronomic parameters

The combined analysis of variance is a useful statistical model that helps express the main effect and estimate the interactions among and within the source of variations as shown in **Table 4**. Significance differences were observed for genotype-by-environment interactions, illustrating the differences in performances of accessions from one location to another, and the agro-pedology of the environments impacted the agronomic characteristics of accessions tested. The mean square of environment for TNPP, LOS, and WOS indicated no significant difference (p > 0.05), while for FPW and HSW, there was a significant difference at p ≤ 0.05. The REP (ENV) showed a mean square that was not significantly different for the traits YPP, HSW, and FPW, with a significant difference for TNPP (p ≤ 0.05), a highly significant difference for LOS (p ≤ 0.01), and very highly significant difference (p ≤ 0.001) for the trait WOS. The mean square of variation among the genotypes was very highly significant (p ≤ 0.001) for YPP, HSW, FPW, and TNPP; highly significant at p ≤ 0.01 for LOS; and significant at p ≤ 0.05 for WOS. Lastly, the G × E interaction mean square showed no significant difference for YPP, FPW, TNPP, and WOS, but highly significant differences (p ≤ 0.01) were observed with LOS and HSW.

**Table 4:** Mean squares of combined analysis of variance of Bambara groundnut yield and yield components in three locations.

SOV	df	YPP	HSW	FPW	TNPP	LOS	WOS
		MS	MS	MS	MS	MS	MS
ENV	2	325.51	1,177.11*	2,284.02*	116.006 ns	3.8417 ns	2.0124 ns
REP (ENV)	6	90.07 ns	83.62 ns	432.27 ns	129.028*	4.4967**	5.6178***
GEN	14	404.62***	1,217.23***	1,300.65***	195.002***	2.9732**	2.8508*
ENV: GEN	28	65.48 ns	342.21**	246.80 ns	75.938 ns	2.2456**	1.2443 ns
Residuals	83	65.23	171.76	320.94	57.916	1.0877	1.3137

SOV, source of variation; df, degree of freedom; MS, mean square; TNP, total number of pods; FPW, fresh pod weight; HSW, hundred seed weight; YPP, yield per plant.

*Significant at p ≤ 0.05; **highly significant at p ≤ 0.01; *** very highly significant at p ≤ 0.001; ns = not significant p > 0.05.

### 3.2 Additive main effects and multiplicative interaction1 biplot

**Figure 1:**
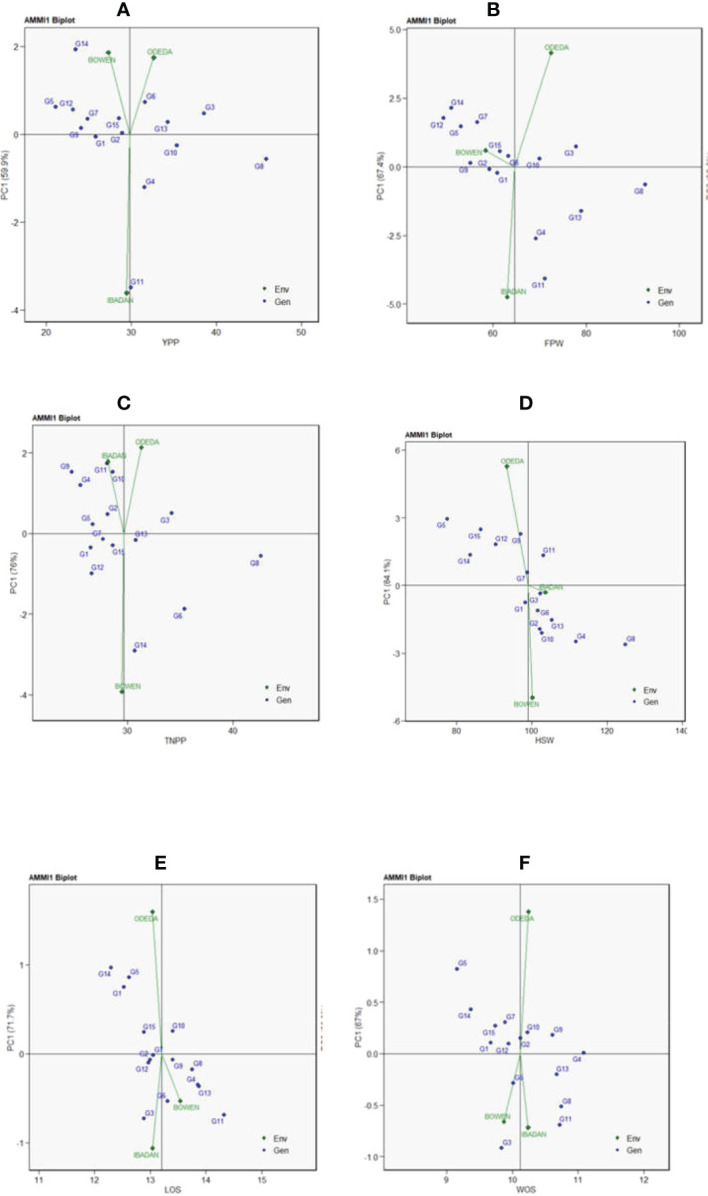
Patterns **(A–F)**. Additive main effects and multiplicative interaction 1 (AMMI 1) biplots based on PC1 illustrating G × E interactions of the 15 Bambara groundnut accessions under three sites: **(A)** yield per plant (YPP), **(B)** fresh pod weight (FPW), **(C)** total no. of pods per plant (TNPP), **(D)** hundred seed weight (HSW), **(E)** length of seeds (LOS), and **(F)** width of seeds (WOS).

Additive main effects and multiplicative interaction 1 (AMMI 1) depicts the biplot abscissa and ordinate illustrating the first principal component analysis (PCA1) term and trait main effects, respectively, in each pattern of **Figure 1**. The first principal component analysis scores for both the environments and the genotypes were plotted against the yield per plant, fresh pod weight, total number of pods, hundred seed weight, and seed length for the environments and genotypes (**Figure 1**: Patterns A, B, C, D, E, and F). The 15 accessions tested in the three locations allowed us to understand the GEI using AMMI through agronomic traits such as yield per plant, fresh pod weight, total number of pods, hundred seed weight, seed length, and width. Similarities and dissimilarities among the 15 accessions were observed on the basis of the GEI study. The PC1 values for the traits observed were 59.9%, 67.4%, 76%, 84.1%, 71.7%, and 67% for the traits measured. To illustrate the effect of each genotype and environment, the AMMI 1 Patterns A, B, C, D, E, and F are shown. It appears that for YPP (Pattern A), genotypes G1, G2, G15, and G9 were closer to the origin, indicating that they recorded almost zero scores on the first PCA1, but genotypes G8, G11, G14, and G3 were far from the origin. For FPW (Pattern B), genotypes G1, G2, G9, G6, and G10 were close to the origin, showing that they had virtually zero scores on the first PCA1, while G4, G13, G11, and G8 were far from the origin. For TNPP (Pattern C), G5, G7, G15, G13, and G1 were nearly close to the origin, i.e., nearly scored zero on the first PCA1, and G14, G8, G6, G9, G4, and G11 are far from the origin. For the HSW (Pattern D), G7, G3, and G1 were closer to the origin, showing that they had practically zero scores on the first PCA1, while G5, G11, G8, and G4 were far from the origin. For LOS (Pattern E), genotypes G12, G2, G7, and G9 were close to the origin, while G11, G14, G5, and G3 were far from the origin. Lastly, in WOS (Pattern F), G1, G12, and G2 were close to the origin, while G3, G11, G8, and G5 were far from the origin. In terms of performance, the mean environments or accessions in AMMI 1 found on the same parallel line in relation to the ordinate were of similar performance; at the same time, those placed at the right side of the center of the axis had higher performance than the Bambara groundnut accessions found at the left-hand side. In Pattern A, G8, G3, G10, G6, G13, G4, and G11 located on the right side of the center of the axis had higher yields than G2, G15, G1, G7, G9, G12, G5, and G14.

### 3.3 Additive main effects and multiplicative interaction2 biplot

**Figure 2:**
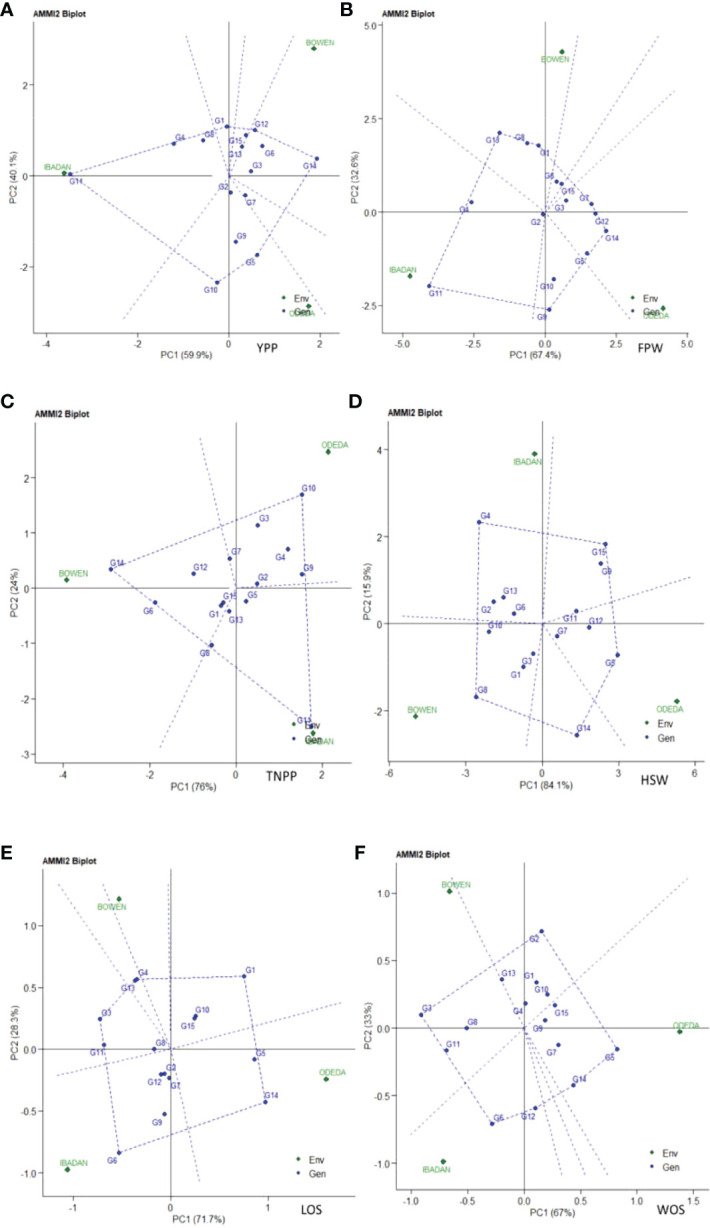
Patterns **(A–F)**. Additive main effects and multiplicative interaction 2 (AMMI 2) biplots based on PC1 and PC2 illustrating G × E interactions of the 15 Bambara groundnut accessions under three sites: **(A)** yield per plant (YPP), **(B)** fresh pod weight (FPW), **(C)** total no. of pods per plant (TNPP), **(D)** hundred seed weight (HSW), **(E)** length of seeds (LOS), and **(F)** width of seeds (WOS).

Additive main effects and multiplicative interaction model 2 (AMMI 2) biplot of Bambara groundnut genotypes are illustrated in **Figure 2**. The first PCA scores for both the environments and genotypes were plotted against the second PCA scores for the environments and genotypes when compared to **Figure 1**, where PCA1 was plotted against agronomic variables. The environmental and genotypic scores of PCA1 and PCA2 were then used to generate the biplot. The AMMI 2 revealed the importance of PCA2 scores along the first PCA in explaining the complexity of GEI involving significant multi-environments and identifying the adaptation of genotypes. In this study, the PCA2 values for the traits observed were 40.1%, 32.6%, 24%, 15.9%, 28.3%, and 33% for yield per plant, fresh pod weight, total number of pods, hundred seed weight, length of seeds, and width of seeds, respectively. It was also observed that the first two PCA interactions accounted for 100% of the G + G × E interaction variation for all the six agronomic traits used for the AMMI model 2 biplot. For yield per plant, G3, G13, G15, G6, G2, and G7 were close to the origin, while G14, G10, and G5 were far from the center. Environment Ibadan had practically zero scores on the first PCA1, while Bowen and Odeda had a high score on the first and second PCAs for yield per plant. For yield per plant, it was observed that genotypes G10 and G5 and Environments Bowen and Odeda had a significant effect on the GE interaction. For fresh pod weight, genotypes G2, G3, G15, and G6 were close to the origin, while G11, G9, and G18 were far from the center. All three environments—Ibadan, Bowen, and Odeda—had a significant effect on the GE interaction. For a total number of pods, genotypes G15, G5, G2, G1, and G13 were close to the origin, while G11, G10, and G14 were far from the center. For YPP, genotype G14 had specific adaptability with Ibadan, G5 had specific adaptation with Odeda, and G12 had specific adaptation with Bowen. For TNPP, G14 showed specific adaptability with Bowen, G11 with Ibadan, and G10 for Odeda. For LOS, G6 demonstrated specific adaptation with Ibadan and G14 with Odeda. For FPW, G11 displayed a specific adaptation with Ibadan, G5 with Odeda, and G6 with Bowen.

### 3.4 GGE biplot

**Figure 3:**
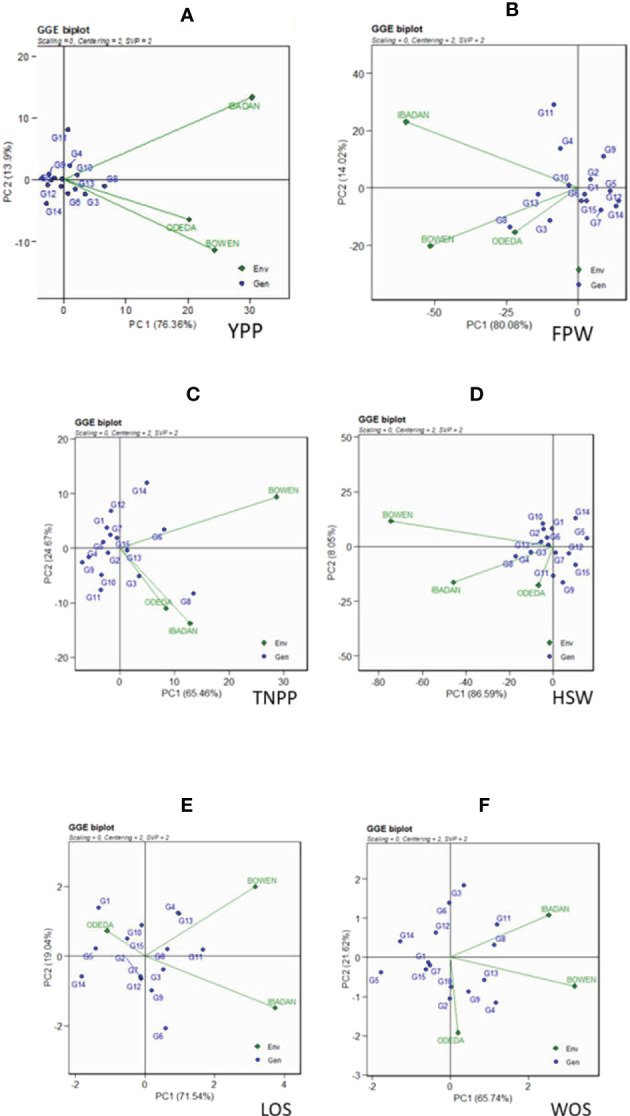
Patterns **(A–F)**. GGE biplots based on PC1 and PC2 showing G × E interactions of the 15 Bambara groundnut accessions under three sites: **(A)** yield per plant (YPP), **(B)** fresh pod weight (FPW), **(C)** total no. of pods per plant (TNPP), **(D)** hundred seed weight (HSW), **(E)** length of seeds (LOS), and **(F)** width of seeds (WOS). The GGE biplots were based on scaling = 0, centering = 2, and singular value partitioning (SVP) = 2.

The GGE biplot is recorded in **Figure 3**: Patterns A, B, C, D, E, and F for YPP, FPW, TNPP, HSW, LOS, and WOS, respectively. Principal component analysis 1 contributed 76.36%, 80.08%, 65.46%, 86.59%, 71.54%, and 65.74% to the total variation. Principal component 2 contributed 13.9%, 14.02%, 24.67%, 8.05%, 19.04%, and 21.62% to the total variation, so principal components 1 and 2 for the traits contributed 90.26%, 94.1%, 87.13%, 94.64%, 90.58%, and 87.36% to the total variation. The GGE biplot is a model that allows us to understand the GEI effects and to detect genotypes targeted for specific environments for their adaptability, as genotypes cannot always win in all environments. Dissimilarity was recorded with environment vectors for the six agronomic traits of the GGE biplot. For YPP, FPW, TNPP, HSP, LOS, and WOS, Ibadan and Bowen showed the longest vectors. The genotypes that were grouped together and facing the direction of the environments yielded above the genotypes that were behind the origin and not grouped with the direction of the environment. For instance, the genotypes that were not grouped together with the environments and were behind the origin were as follows: in YPP, genotypes G12, G9, G1, and G14; in FPW, genotypes G9, G2, G1, G5, G12, G7, and G14; in TNPP, G12, G1, G7, G4, G9, G10, and G11; in HSP, G14, G5, G9, G15, G7, and G12; in LOS, G14, G5, G2, G7, and G12; and in WOS, G5, G14, G6, G12, G15, and G7. In contrast, the following were grouped together with the environment: in YPP, G6, G3, G8, G10, G4, G13, and G11; in FPW, G11, G4, G10, G13, G8, and G3; in TNPP, G14, G5, G13, G3, and G8; in HSP, G10, G2, G13, G8, G4, G11, and G3; in LOS, G10, G15, G4, G13, G11, G6, G9, and G3; and in WOS, G11, G8, G13, G9, G4, and G3.

### 3.5 Ranking genotypes

**Figure 4:**
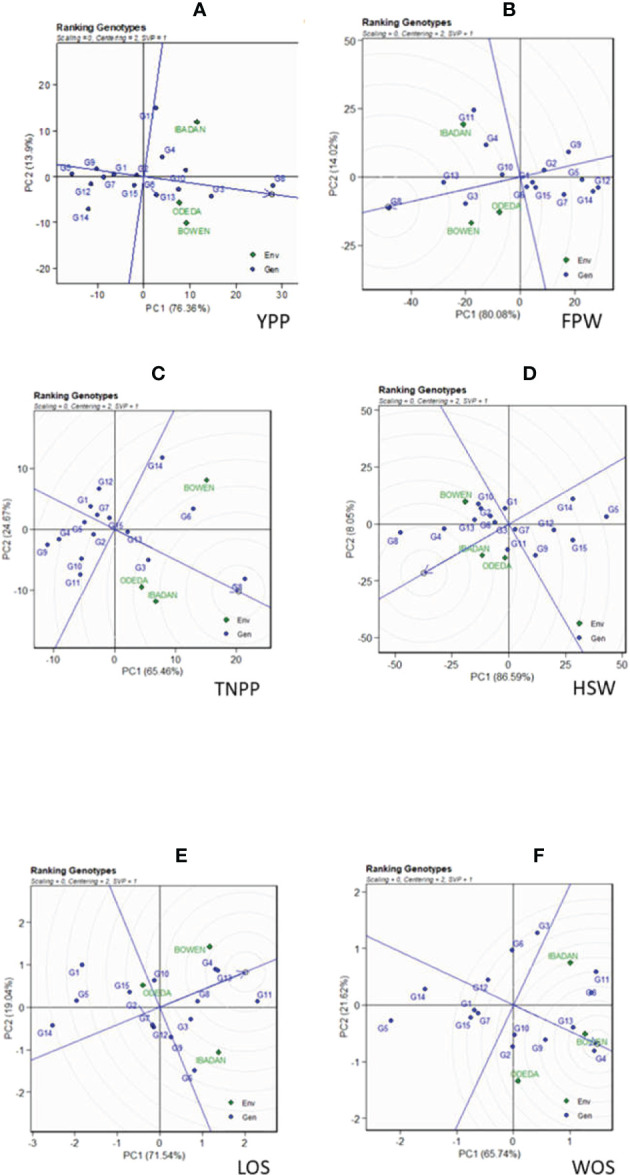
Patterns **(A–F)**. Ranking genotypes based on PC1 and PC2 showing G × E interactions of the 15 Bambara groundnut accessions under three sites: **(A)** yield per plant (YPP), **(B)** fresh pod weight (FPW), **(C)** total no. of pods per plant (TNPP), **(D)** hundred seed weight (HSW), **(E)** length of seeds (LOS), and **(F)** width of seeds (WOS). The ranking genotypes were based on scaling = 0, centering = 2, and singular value partitioning (SVP) = 1.

**Figure 5:**
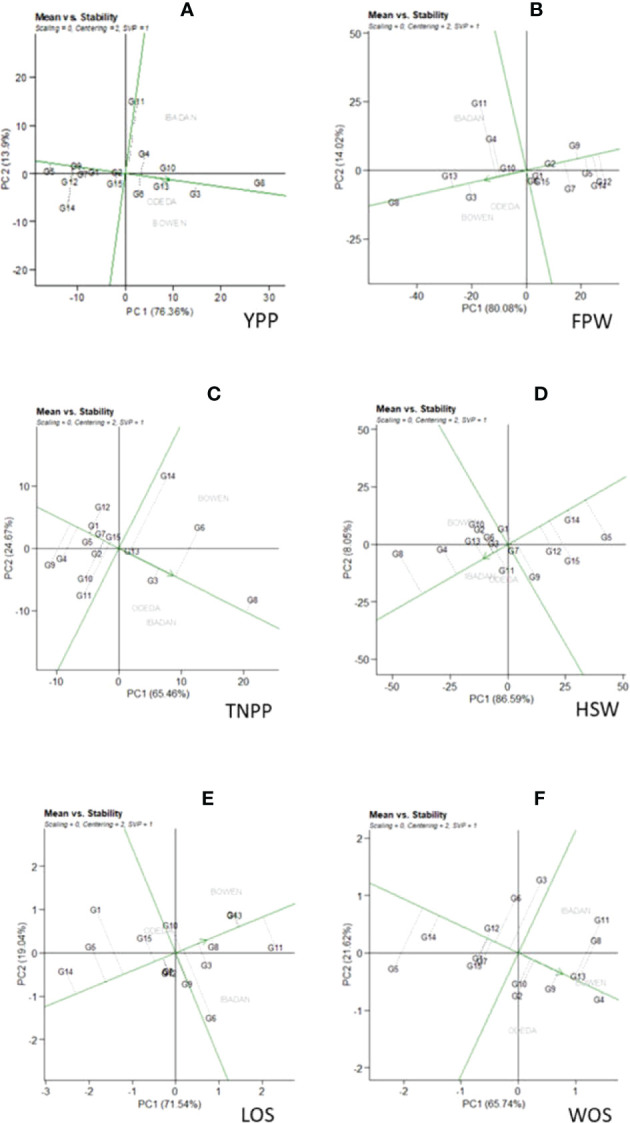
Patterns **(A–F)**. Mean *vs.* stability based on PC1 and PC2 showing G × E interactions of the 15 Bambara groundnut accessions under three sites: **(A)** yield per plant (YPP), **(B)** fresh pod weight (FPW), **(C)** total no. of pods per plant (TNPP), **(D)** hundred seed weight (HSW), **(E)** length of seeds (LOS), and **(F)** width of seeds (WOS). The mean *vs.* stability was based on scaling = 0, centering = 2, and singular value partitioning (SVP) = 1.

The genotype ranking is shown in **Figure 4** (Patterns A, B, C, D, E, and F). The biplot allowed us to identify the best and ideal genotype among the 15 tested genotypes. An ideal genotype is always localized into the innermost circle and virtually closer to the head of the arrow at the center of the circular ring (**Figure 4**: Patterns A, B, C, D, E, and F). The only genotype that fell inside the inner circle was G8; the genotypes next to the ideal inner circle were G3 and G10, followed by G13, G6, G4, and G11 for YPP (**Figure 4**: Pattern A). Similarly, for FPW, the only genotype was G8, while G13, G3, G11, G10, and G4 were next to the ideal genotype (**Figure 5**: Pattern B). For TNPP, the only genotype was G8, while G6, G3, G13, and G14 followed the only ideal genotype (**Figure 4**: Pattern C). For HSW, there were no genotypes inside the inner circle, but the genotypes next to the inner cycle were G8, G4, G13, G11, G10, G3, G2, and G6 (**Figure 4**: Pattern D). For LOS, there were no genotypes inside the inner circle, but genotypes G11, G13, G4, G8, G10, G9, and G6 were next to the inner cycle (**Figure 4**: Pattern E). For WOS, the only genotype that was placed inside the inner cycle was G4, while G8, G11, G13, G9, G10, and G2 were next to the ideal genotype (**Figure 4**: Pattern F). However, the following were very far from the arrowhead in the plot: for YPP, G2, G15, G1, G7, G9, G14, G12, and G5; for FPW, G6, G15, G1, G2, G7, G9, G5, and G14; for TNPP, G10, G15, G2, G11, G7, G12, G1, G5, G4, and G9; for HSP, G7, G1, G9, G12, G15, G14, and G5; for LOS, G7, G2, G12, G3, G3, G15, G5, G1, and G14; and lastly, for WOS, G6, G12, G7, G3, G15, G1, G14, and G5. Therefore, the ranking of genotypes based on the ideal genotype for yield per plant was G8 > G3 > G10 > G13 > G6 > G4 > G11 > G2 > G15 > G1 > G7 > G9 > G14 > G12 > G5 (**Figure 4**: Pattern A).

### 3.6 Mean *vs.* stability

In **Figure 5**, Patterns A, B, C, D, E, and F show the mean *vs.* stability of the genotypes evaluated. The abscissa and the ordinate of the average environment coordinate (AEC) are the two lines passing through the origin of the biplot on the basis of singular value partitioning (SVP = 1). The average environment coordination abscissa showed a single direction arrow indicating the ideal genotype main effect, while the average environment coordination ordinate with double arrows though outside the biplot illustrated greater GEI effect and lower stability. The ordinate divides the genotypes into two: those that yielded above and below average. The following are the genotypes toward the arrow that yielded above the average means: G8, G3, G13, G6, G4, and G10 (Pattern A); G8, G13, G3, G10, G4, and G11 (Pattern B); G8, G6, G3, G13, and G14 (Pattern C); G8, G4, G13, G3, and G10 (Pattern D); G11, G4, G3, G8, G6, and G10 (Pattern E); G4, G13, G9, G10, G2, G10, and G11 (Pattern F). In contrast, the following fell below the average means: G5, G12, G14, and G7 (Pattern A); G12, G14, G5, and G7 (Pattern B); G9, G4, G5, G1, and G12 (Pattern C); G5, G14, G15, and G12 (Pattern D); G14, G5, G1, and G15 (Pattern E); G5, G14, G15, and G12 (Pattern F). The abscissa, therefore, points toward increased order of genotype performance. Based on YPP, FPW, TNPP, HSP, LOS, and WOS, G8, G8, G8, G8, G11, and G4 were the highest genotypes, while G5, G12, G9, G5, G14, and G5 were the lowest genotypes. The projection on the abscissa toward the ordinate of the AEC is a measure of stability, so a genotype with zero projection or very short direction from the ordinate is considered the most stable, while a genotype with the longest projection from the abscissa is unstable. Based on YPP, FPW, TNPP, HSP, LOS, and WOS, G8, G8, G13, G7, G8, and G4 had the shortest projection, while G11, G11, G14, G9, G6, and G3 had the longest.

### 3.7 Estimated values of the average of the stability and mean performance of 15 genotypes

**Figure 6:**
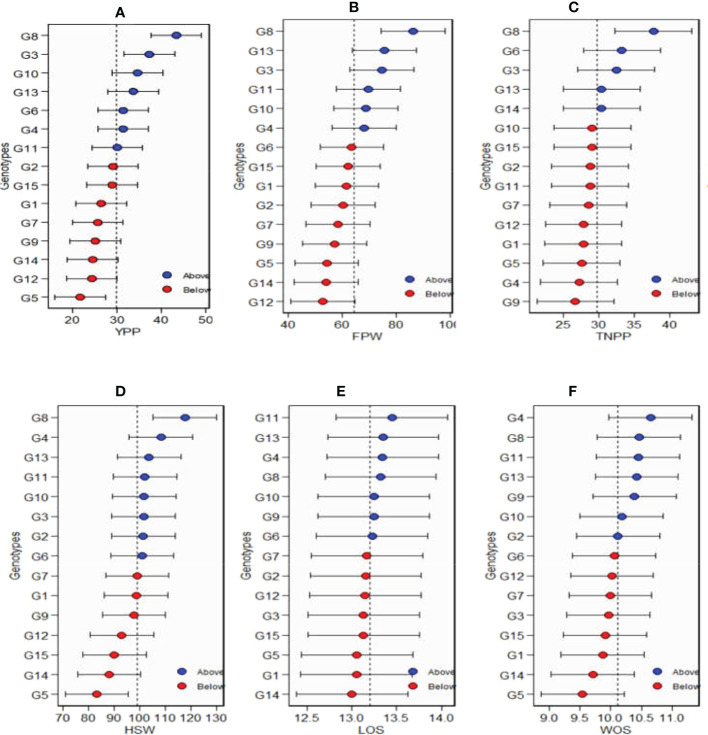
Estimated values of average of the stability and mean performance for 15 Bambara groundnut genotypes: **(A)** yield per plant (YPP), **(B)** fresh pod weight (FPW), **(C)** total no. of pods per plant (TNPP), **(D)** hundred seed weight (HSW), **(E)** length of seeds (LOS), and **(F)** width of seeds (WOS).

The estimated values of mean performance for 15 Bambara groundnut genotypes are shown in **Figure 6**. The genotypes in blue circles were the best in terms of their mean performances, while the red circles illustrate the genotypes with performances below the average, and the genotypes at the bottom were the poorest (**Figure 6**). The horizontal error bars shown in **Figure 6** for each genotype denote the 95% confidence interval for the estimated values for yield per plant, fresh pod weight, total number of pods, hundred seed weight, length of seeds, and width of seeds predicted. For yield per plant, among the tested genotypes in the three locations, G8 had the highest mean performance followed by G3, G10, G8, G4, and G11, which were also above the mean, while G2, G15, G1, G7, G9, G14, G12, and G5 recorded the lowest mean performances. Similarly, for fresh pod weight, G8 had the highest mean performance followed by G13, G3, G11, G10, and G4, while the lowest was recorded with G12. For TNPP, G8 had the highest mean performance followed by G6, G3, G13, and G14, while G9 had the poorest. G11 and G4 took the lead as genotypes with the highest mean performances for LOS and WOS, respectively.

### 3.8 Which-won-where

**Figure 7:**
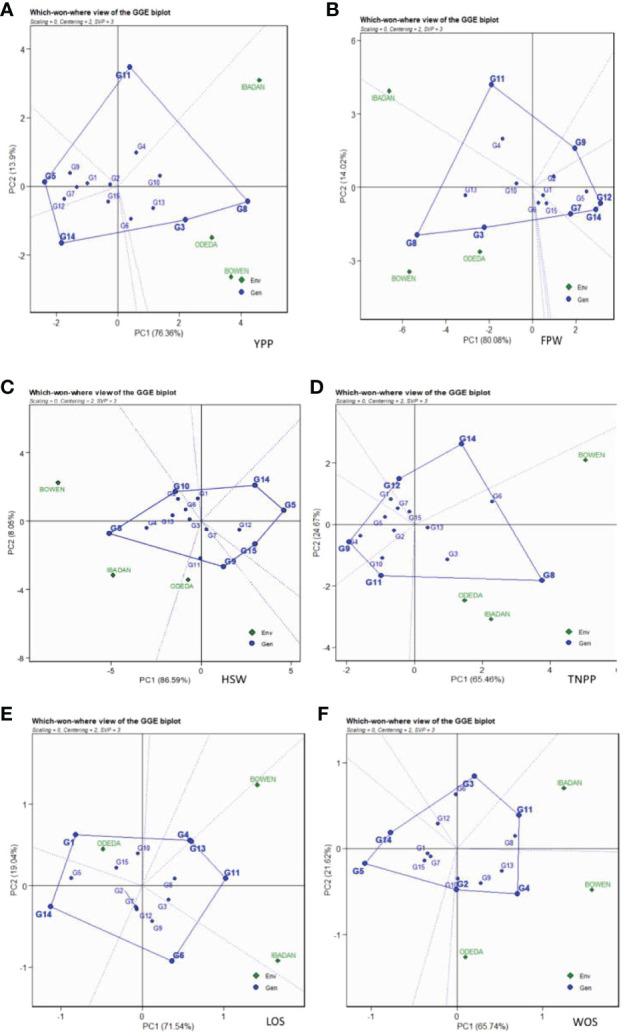
Patterns **(A–F)**. Polygon views of GGE biplot for which-won-where analysis of 15 accessions under the effects of genotypes-by-environment interactions: **(A)** yield per plant (YPP), **(B)** fresh pod weight (FPW), **(C)** hundred seed weight (HSW), **(D)** total no. of pods per plant (TNPP), **(E)** length of seeds (LOS), and **(F)** width of seeds (WOS). The GGE biplot polygons were based on scaling = 0, centering = 2, and singular value partitioning (SVP) = 3.

The GGE biplot for which-won-where (**Figure 7**: Patterns A, B, C, D, E, and F) shows that the first and second component analyses (PCA1 and PCA2) accounted for 90.26%, 94.08%, 90.13%, 94.64%, 90.58%, and 87.36% of the total variation for YPP, FPW, HSW, TNPP, LOS, and WOS, respectively. The test environments fell into one of the five sectors, two of the seven sectors, two of the six sectors, one of the five sectors, two of the six sectors, and two of the six sectors outlined on the polygon view for YPP, FPW, HSW, TNPP, LOS, and WOS, respectively. Thus, the mega-environments were identified for each trait. There were one mega-environment for YPP, two mega-environments for FPW, and two mega-environments for HSW with Bowen and Ibadan grouped together in a mega-environment, while Odeda is in the second mega-environment. There were one mega-environment for TNPP and two mega-environments for LOS with Bowen and Ibadan grouped together in a mega-environment, while Odeda is in the second mega-environment. Lastly, there were two mega-environments for WOS with Bowen and Odeda grouped together in a mega-environment, while Ibadan is in the second mega-environment. The vertex genotype was also identified as G8 and G3 for YPP; G8 and G3 for FPW; G8 and G2 for the first mega-environment and G11 and G9 for the second mega-environment in HSW; G8 and G6 for TNPP; G4, G11, and G13 for Bowen and Ibadan mega-environment; G1 for Odeda mega-environment in LOS; lastly, G4 and G2 for WOS. Some vertex genotypes were observed to not fall into sectors containing the test environment, for instance, G5, G14, and G11 (Pattern A); G9, G12, and G7 (Pattern B); G15, G9, and G14 (Pattern C); G11, G4, and G12 (Pattern D); G6 and G14 (Pattern E); G5, G14, and G3 (Pattern F).

### 3.9 Discriminativeness *vs.* representativeness

**Figure 8:**
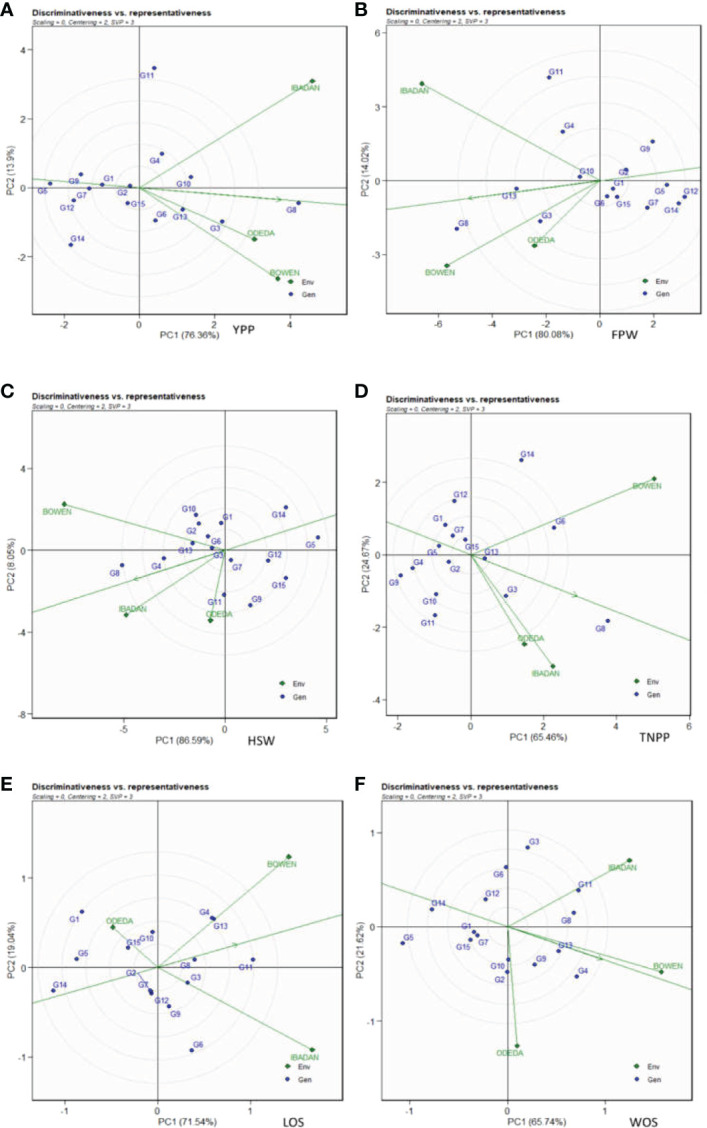
Discriminativeness and representativeness GGE biplot of 15 accessions under the effects of genotypes-by-environment interactions: **(A)** yield per plant (YPP), **(B)** fresh pod weight (FPW), **(C)** total no. of pods per plant (TNPP), **(D)** hundred seed weight (HSW), **(E)** length of seeds (LOS), and **(F)** width of seeds (WOS). The GGE biplot polygons were based on scaling = 0, centering = 2, and singular value partitioning (SVP) = 3.

In **Figure 8**, Patterns A, B, C, D, E, and F illustrate the “discriminativeness *vs.* representativeness” of the GGE biplot study. The vector length for each environment revealed the discriminatory ability of the environment, while the angle formed by each vector with the abscissa denotes representativeness. The shortest vector for Patterns A, B, C, D, E, and F was Odeda, while the longest vector for Patterns A, B, and E was Ibadan and for Patterns C, D, and F was Bowen. The angles the environment formed with the abscissa line were also recorded. For YPP, the shortest angle was formed by Odeda. For FPW, HSW, WOS, and LOS, the shortest angle was formed by Bowen. For TNPP, the shortest angle was formed by Ibadan. The longest angles formed by the environment were also observed to be Ibadan for YPP, while for FPW, TNPP, HSW, LOS, and WOS, they were formed by Odeda. The biplot identified the environments that were closest to the AEC. The environments were Odeda for YPP, Bowen for FPW, Ibadan for HSW, Ibadan for TNPP, Bowen for LOS, and Bowen for WOS.

### 3.10 Ranking environments

**Figure 9:**
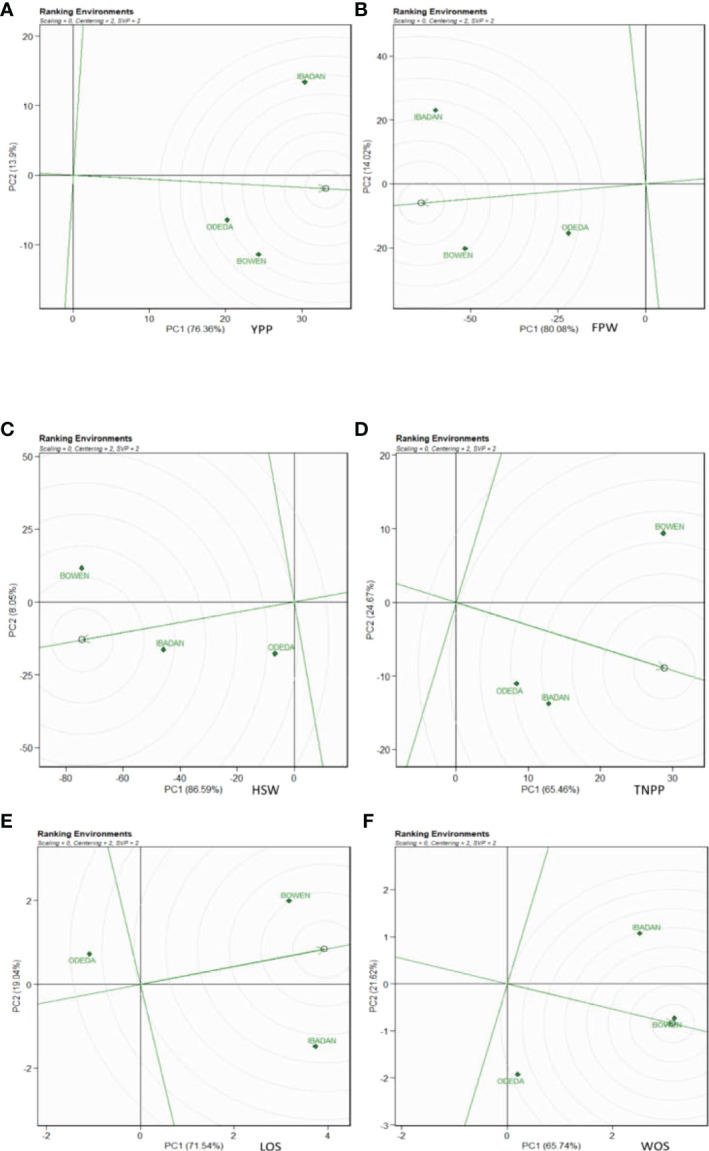
Ranking environments with the ideal environments for the growth of Bambara groundnut with respect to **(A)** yield per plant (YPP), **(B)** fresh pod weight (FPW), **(C)** total no. of pods per plant (TNPP), **(D)** hundred seed weight (HSW), **(E)** length of seeds (LOS), and **(F)** width of seeds (WOS). The GGE biplot polygons were based on scaling = 0, centering = 2, and singular value partitioning (SVP) = 2.

In **Figure 9**, Patterns A, B, C, D, E, and F show the ranking biplot for comparison of the environments with the ideal environment. This figure identifies the most appropriate and most inappropriate environments. Based on this, the best environment is the one that has the closest distance from the ideal environment (concentric circles), and the most undesired one is the environment with the furthest distance from the ideal environment. For the traits YPP, FPW, HSW, LOS, and WOS, Bowen was closer to the putative ideal environment followed by Ibadan, and Odeda was seen to have had the furthest distance to the putative ideal environment. For TNPP, Ibadan was closer to the putative ideal environment followed by Bowen, and Odeda was the farthest from the ideal environment.

### 3.11 Relationship among environments

**Figure 10:**
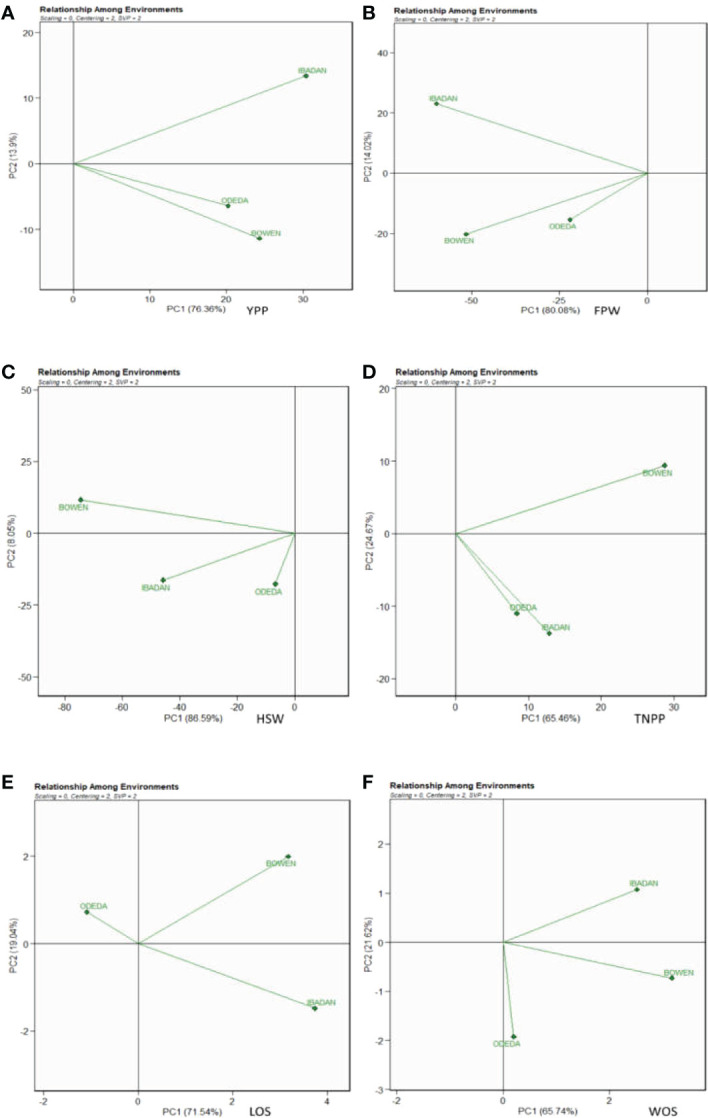
Patterns **(A–F)**. The relationship among environments tested for the production of 15 Bambara groundnut accessions with respect to **(A)** yield per plant (YPP), **(B)** fresh pod weight (FPW), **(C)** total no. of pods per plant (TNPP), **(D)** hundred seed weight (HSW), **(E)** length of seeds (LOS), and **(F)** width of seeds (WOS). The GGE biplot polygons were based on scaling = 0, centering = 2, and singular value partitioning (SVP) = 2.

The evaluation of the test environments is vital in the discrimination and representation of ideal and stable genotypes and environments for the study of GEI in multi-environment trials. In **Figure 10**, Patterns A, B, C, D, E, and F characterize the possible relationship among environments tested for the production of 15 Bambara groundnut accessions in this study. The biplot accounted for 76.36% (PC1) and 13.9% (PC2) for YPP, 80.08% (PC1) and 14.02% (PC2) for FPW, 86.59% (PC1) and 8.05% (PC2) for HSW, 65.45% (PC1) and 24.67% (PC2) for TNPP, 71.54% (PC1) and 19.04% (PC2) for LOS, and 65.74% (PC1) and 21.62% (PC2) for WOS of G + G × E interaction variation across the tested environments. In Patterns A, B, C, and D, the cosine angle of vectors for Bowen and Ibadan, Ibadan and Odeda, and Bowen and Odeda were <90°, indicating positive correlations among them. Thus, they can be classified into one group. In Pattern E, Bowen and Ibadan were <90°, and Ibadan and Odeda and Bowen and Odeda were >90°, indicating that these locations were correlated negatively. In Pattern F, Bowen and Ibadan and Bowen and Odeda were <90°, while Ibadan and Odeda were >90°, which indicate a negative correlation between them. The size in lengths of the environment vectors for the traits was also observed. Based on our findings, we categorized the lengths into three groups. Group 1 includes the long vectors, which include Ibadan (YPP), Bowen and Ibadan (FPW), Bowen (HSW), Bowen (TNPP), Ibadan (LOS), and Bowen (WOS). Group 2 includes the medium vectors, which are Bowen (YPP), Ibadan (HSW), Ibadan (TNPP), Bowen (LOS), and Ibadan (WOS). Group 3 includes the short vector, which is Odeda for YPP, FPW, HSW, TNPP, LOS, and WOS.

## 4 Discussion

To guarantee food security, there is a need to increase crop productivity through plant improvement and breeding programs. Multi-environment experiments, commonly called genotype-by-environment interactions, constitute the important steps in plant improvement and breeding programs. It is then the responsibility of breeders to increase crop productivity and make sure that the growing human population is properly fed. Therefore, AMMI and GGE models are employed in the current study to select genotypes with high adaptability and stability.

### 4.1 Combined analysis of variance for agronomic traits

The variance evenness of experimental errors was examined in which the results confirmed the homogeneity of such errors. Hence, a combined analysis of variance for agronomic traits of interest was carried out, and the results indicated that the location, genotype, and genotype × location effects range from not significant to highly significant and then to very highly significant for the entire agronomic traits observed in this study. The significance of the environment effect revealed that environments differed in terms of agronomic traits of genotypes, while the non-significance of some locations for certain traits of interest implies that the locations did not vary. The significance of the genotype-by-environment interaction shows that the genotypes’ agronomic traits vary from one location to another, while the non-significance of genotype-by-environment interaction for some traits showed that the traits did not diverge from one location to another. In the corn hybrid study for grain yield, Haruna et al. (2017); Azrai et al. (2022), and Hudson et al. (2022) reported findings in multi-environment trials where the main proportion of total phenotypic variation was ascribed to the environment and comparatively minor sources of variation to genotype, while in this present study, a larger proportion of variation was attributed to the genotypes. This study revealed very highly significant differences in genotypes and significant differences in environments, which could be credited to environmental conditions, the genetic diversity of the genotypes, and the effects of the interaction between the genotypes and environments. The highly significant GEI for some agronomic traits could be explained by the heterogeneity of the nature of the multi-environments tested with the different genetic makeup of the genotypes planted, and similar results were reported by many researchers (Yan and Kang, 2003; Andrade et al., 2016; Olanrewaju et al., 2021; Khan et al, 2021). Nevertheless, the variance component analysis is insufficient to elucidate all the attributes of the genotype-by-environment interaction. Subsequently, more statistical tools and models could be more beneficial and prolific in describing and comprehending the more the GEI (Oladosu et al., 2017). The genotype-by-environment interaction effect primarily highlights the fact that genotypes responded inversely to various locations, emphasizing the need for genotype assessment in diverse environments. Similarly, the GEI study is a means through which plant breeders select the ideal and best genotypes for commercial purposes, which is not without challenges. The partitioning of the environment indicates that the sources of variation could be due to experimental sites, climatic conditions of the sites, or growing season of the crops (Oladosu et al., 2017; Khan et al., 2021; Azrai et al., 2022).

### 4.2 AMMI 1 biplots

AMMI is one of the important models that evaluate the significance of the G × E interaction of agronomic traits in multi-environment trials. The AMMI model allows us to comprehend the interrelationship between genotypes and the environments involved. AMMI model 1 biplot is one of the many versions of AMMIs, basically for identifying high potential yield and stability **(** Olivoto et al., 2019). Kılıç (2014) reported that in AMMI 1 when the mean environments and genotypes are placed on the same lines that are parallel to the ordinate, it means that they have almost the same performance, but when the genotypes are placed on the right side of the center of the biplot, they show higher yield than those genotype at the left-hand side. These results are similar to our findings where all the genotypes like G8, G3, and G13 located on the right side were the best genotypes for YPP, TNPP, HSW, FPW, LOS, and WOS. Genotypes that together show the same adaptability while environments that are together influence the genotypes similarly. In this study, the results of AMMI analysis indicated that the AMMI model fits the data well and justifies the use of AMMI 2. This made it possible to construct the biplot and calculate genotype and environment effects (Gauch and Zobel, 1996; Vargas and Crossa, 2000; Yan and Hunt, 2001; Kaya et al., 2002). Also, the closer the genotypes are to the origin, the less interactive they are, and the farther the genotypes are to the origin, the more interactive they are. For instance, in YPP (Pattern A), G2 was closer to the origin, and therefore, it is less interactive, while G8 is far from the origin, and therefore, it is highly interactive. This interpretation follows for the other genotypes that were observed to be close or far from the origin for other traits measured. The graph shows that the genotypes on the right side of the perpendicular, i.e., (Pattern G) Odeda, G3 and G6 and Bowen, and G8 and G4 genotypes are less affected by G × E interaction. The closer the interaction principal component axis (IPCA) Mur scores to zero, the more stable the genotypes are across their testing environments (Carbonell et al., 2004). In Patterns A, B, C, D, E, and F, genotypes G2, G5, G13, G7, and G12 were close to the center point and indicated that they are stable across the environments. When the PCA1 score for a genotype or environment is near zero, there is a small interaction impact; on the contrary, if a genotype and environment achieve the same sign on the PCA axis, there is a positive interaction; otherwise, there is a negative interaction. Reports published by Mogale (2018) and Khan et al. (2021) are comparable to our findings on Bambara groundnut and Oladosu et al. (2017) in rice.

### 4.3 AMMI model 2

The AMMI model 2 biplot is produced to make use of the environmental and genotypic scores of the first two AMMI components as reported by Vargas and Crossa (2000) and Kılıç (2014). The IPCA1 and IPCA2 scores help to understand the function of genotype-by-environment interaction and the adaptability of the genotypes in the test environments. Our observation about the first two PCA interactions presenting 100% of the G + G × E interaction variation for all the six agronomic traits with AMMI model 2 biplot could be explained by the fact that only the two IPCAs were involved in the total variation. Our findings are in agreement with those of Gauch and Zobel (1996) and Khan et al. (2021), who reported that the first two PCAs are enough for the projection of the AMMI model, though some researchers proposed the first four PCs in the multi-environment trial (Sivapalan et al, 2000).

AMMI 2 is made of four quadrants from the center of the biplot (0, 0) when considering the vertical and horizontal lines. The nearer the genotypes to the ordinate axis, the more they show their general adaptation, while the farther the genotypes, the more they express specific adaptability to the environments. In this study, the stable and unstable genotypes were detected through AMMI 2. The findings of the current research works are similar to those of Purchase (1997), who reported that the nearer the genotypes score to the center of the AMMI 2 model biplot, the more stable they are. The same results were obtained by Kılıç (2014), and Khan et al. (2021). Moreover, the genotypes placed in a quadrant with the environment are considered the winning genotypes in that environment. For YPP, G14 and G5 are winning genotypes in Odeda.

### 4.4 GGE biplot

AMMI model 1 biplot, AMMI MODEL 2 biplot, and GGE biplot are the three common models used in studying multiple environment trials. Many features worthy of mention, which characterize the three models, are the PCAs, the center of the biplots, vectors, vertical and horizontal lines, the polygon and polygon indicators or markers, and the polygon vertex. The discriminativeness and representativeness abilities of the GGE biplot make it more sophisticated than AMMI (Sharma et al, 2020). Apart from the representativeness and discriminativeness capabilities of the GGE model, its “which-won-where” pattern and the stability *vs.* mean performance of the genotypes in the multi-environment trials (Yan et al., 2000; Angelini et al, 2019) were also revealed. Thus, AMMI has the ability to identify GEI in many environments with different characteristics by using a biplot model. The GGE biplot allows us to comprehend better the complex GEI and will aid researchers in better understanding complicated GE interactions in multi-environment for newly developed varieties, hybrids, and agronomic trials (Luo et al., 2015; Khan et al., 2021). Moreover, the GGE model evaluates the performance of accessions subjected to abiotic and biotic stress, ideal accession/genotypes, mega-environment from many environments, and testing locations (Karimizadeh et al., 2013). GGE shows directly the genotype effects, which cannot be carried out with the AMMI 2 model but which decomposes G × E interaction effects in the principal component analysis (Karimizadeh et al., 2013; Khan et al., 2021). GGE model has an advantage over AMMI for detecting stable and high-yielding genotypes, genotypes with stable yield across multi-environments, and genotypes that perform in each location. The discrepancies in arable lands due to pedoclimatic conditions, therefore, lead to the GEI effect, thus causing crops to not perform uniformly in the environments. For these reasons, plant breeders give much attention to GEI studies to provide lasting solutions to this problem by applying AMMI and GGE approaches (Eberhart and Russell, 1966; Crossa, 1990; Alizadeh et al., 2017; Oladosu et al., 2017; Khan et al., 2021). The GGE biplot helps understand GEI effects and identify genotypes for specific environments and adaptation and stability, as genotypes cannot perform or win in all environments and are always due to the GEI effect. In the present study, the 15 Bambara groundnut accessions responded differently in the diverse environments of Bowen, Ibadan, and Odeda. Therefore, the GEI effects are based on the 15 genotypes and the three environments assessment using representativeness and discriminativeness abilities, stability *vs.* mean performance, “which-won-where” pattern, ranking genotypes, and relationship among environments, as explained in the following paragraphs.

### 4.5 Mean *vs.* stability

The GGE biplot of the mean performance and stability of 15 Bambara groundnut stability was assessed through the two PCAs and the projections from the abscissa toward the AEC ordinates. The size of the projection determines the stability of the genotypes; thus, the longer the projection, the more unstable the genotype, and the shorter the projection, the more stable the genotype (Yan et al, 2007). The stability analysis of yield complements the genotype ranking analysis in determining the adaptability and stability of genotypes based on their agronomic performances across the test locations (Azrai et al., 2022). In this study for YPP, FPW, TNPP, HSP, LOS, and WOS the shortest projection was identified as G8, G8, G13, G7, G8, and G4, respectively, and therefore identified as the most stable. G11, G11, G14, G9, G6, and G3 had the longest projection and were therefore identified as the most unstable. The GGE biplot determines the desired genotypes on the basis of the average yield and relative stability. The analysis identified genotype G8 as a good combiner of high yield and stability; the ANOVA showed that the G8 was the top performer in the environments. This implies that the genotype was well-buffered and thus circumvented fluctuations in performance across environments (Heinrich et al., 1983). Becker and Leon (1988) identified a successful cultivar as one that possesses high and stable yield potential over a wide range of environmental conditions. Genotypes G11 (YPP), G11 (FPW), G14 (TNPP), G9 (HSP), G6 (LOS), and G11 (WOS) all performed above average but were not stable, indicating that they were inconsistent in performance and therefore unpredictable. G14 (YPP), G12 (FPW), G9 (TNPP), G15 (HSP), G1 (LOS), and G5 (WOS) were worse off as they were identified to be unstable and yielded below average. Similar result trends were observed by Shim et al. (2015) and Khan et al. (2021). The influence of GEI on genotypes requires multi-environment trials as a paramount step in plant breeding. The presence of the interaction among genotypes and environment imposes that the decision on desired genotypes could be not only based on the mean performance of the traits but also associated with the stability of the genotypes to avoid considerable commercial losses. Genotype G8 has the best combined high yield and stability and therefore was considered the most desirable for the three locations used.

### 4.6 Ranking genotypes

In the ranking of genotypes, we detected the ideal genotypes in contrast to other genotypes evaluated. The ideal genotypes identified were followed by the second-best genotypes due to their closeness to the concentric circles based on each agronomic trait studied. However, genotypes that were very far from the arrowhead implied that they were poorly yielding. Commonly, an ideal genotype is always placed into the innermost circle and nearer the head of the arrow at the center of the circular ring. The genotypes placed in the inner circle were highly appropriate as compared to the genotypes of the outer circle. However, in some cases, no genotype was positioned inside the inner circle; consequently, genotypes next closer to the inner circle are considered to be ideal ones (Oladosu et al., 2017; Khan et al., 2021). Thus, the following are regarded as ideal genotypes across the tested environment because they were positioned closer to the center of the biplot origin, indicating that they are stable genotypes: genotypes G2 and G6 for YPP; genotypes G1 and G5 for FPW; genotypes G15 and G13 for TNPP; genotypes G3 and GG7 for HSW; genotypes G7 and G12 for LOS; genotypes G10 and G7 for WOS. For an effective selection, an ideal and best genotype is required to have both high mean and stability properties (Yan and Tinker, 2006). A ring at the head of the arrow on the horizontal AEC abscissa axis generally represents an ideal genotype (Oladosu et al., 2017), and additionally, the best genotype should be positioned in the small circle on the AEC abscissa line. Plant breeders used data from agronomic performance during evaluations on the basis of mean performance and stability to choose genotypes best suited to a specific environment within a multi-environment (Kouassi and Bi, 2010), while genotypes close to the ideal genotype were also more promising or appropriate. Therefore, the genotype ranking for WOS (**Figure 6**: Pattern F) is G4 > G8 > G11 > G13 > G9 > G10 > G2 > G6 > G12 > G7 > G3 > G15 > G1 > G14 > G5. It also applies to the other traits measured. Oladosu et al. (2017) found similar findings across 10 settings as evidence of our result.

### 4.7 Which-won-where

The “which-won-where” is also one of the important components of the GGE biplot for the GEI analysis. The “which-won-where” biplot identifies mega-environment disparity for an environment suitable for the genotypes’ adaptability, the best genotypes in each mega-environment, and the ideal genotype with high agronomic performance and stability (Gauch and Zobel, 1997; Yan, 2001). From the GGE biplot, it was shown that accessions were well adapted in each environment and confirmed the presence of interaction differentiation between genotypes and environments. The detected mega-environment for each agronomic trait allows us to select the outstanding accessions for that very trait in that environment, especially the accessions at the corners of the polygons in the biplot. Thus, the vertex genotypes were identified, indicating their performance and adaptability in the mega-environment. This infers that the vertex genotypes were most favored by the environments, and therefore, they were the most responsive and exceptional genotypes when considering their potential yield in their respective mega-environments (Hashim et al., 2021). However, vertex genotypes with no environment in the sector are not desirable because of their poor performance across the environments (Khan et al., 2021). The accessions used in the present study showed different agronomic trait values across the environments, which indicate the cross-over GEIs. Similar results were obtained by many researchers including Nehe et al. (2019) and Kendal (2019). The environmental adaptation of varieties is very paramount in comprehending their genetic basis, which is only achieved through genotype-by-environment interactions (Hudson et al., 2022).

### 4.8 Discriminativeness *vs.* representativeness analysis

The discriminativeness *vs.* representativeness pattern of the GGE biplot pinpoints how the best environment can be informative and representative. The two concepts focus on environments in terms of their ability to detect the best genotypes (discriminativeness) and to adequately represent the test environments (representativeness) (Yan and Tinker, 2006; Oladosu et al., 2017; Khan et al, 2021). The use of multi-environmental trials is very beneficial because it helps to avoid overestimation of heritability and genetic variance, which are always observed with one location experiment (Azrai et al., 2022). Our study revealed the environments closely related, demonstrating the discriminativeness ability and the representativeness of the test environments. The longer the vector of an environment, the higher its capability to discriminate among genotypes, while the shorter the angle formed with the abscissa, the more it is representative. The present investigation revealed the following trends in terms of the length of the test environments: the long vectors, which include Ibadan (YPP), Bowen and Ibadan (FPW), Bowen (HSW), Bowen (TNPP), Ibadan (LOS), and Bowen (WOS), which were in Group 1. Group 2 includes the medium vectors, which are Bowen (YPP), Ibadan (HSW), Ibadan (TNPP), Bowen (LOS), and Ibadan (WOS). Group 3 includes the short vectors, which are made up of Odeda for YPP, FPW, HSW, TNPP, LOS, and WOS. The longest vector for YPP, FPW, and LOS was Ibadan and for patterns HSW, TNPP, and WOS was Bowen. For YPP, the shortest angle was formed by Odeda. For FPW, HSW, WOS, and LOS, the shortest angle was formed by Bowen, while for TNPP, the shortest angle was formed by Ibadan.

### 4.9 Ranking environments

Bowen and Ibadan were observed as the best environments for YPP, FPW, HSW, LOS, and WOS, while Odeda was observed as the most undesired location for these traits. Ibadan and Bowen were preferred to Odeda for the agronomic performance of TNPP. Our findings showed that Bowen, Ibadan, and Odeda constitute different multiple environments necessitating different Bambara ground varieties for higher yield. These are in agreement with those of Yan and Tinker (2006) and Khan et al. (2021). It is reported that the environment impacts the genotypes due to weather (rainfall and temperature) that cannot be predicted and the soil hosting the genotypes (Lin and Binns, 1994). Hence, the order of ranking the environments for YPP, FPW, HSW, LOS, and WOS is Bowen > Ibadan > Odeda, while for TNPP, the environment ranking is Ibadan > Bowen > Odeda.

### 4.10 Relationship among environments

The current study was carried out in two distinct agro-ecological zones in Nigeria: Southern Guinea Savanna is defined by Ologuneru in Ibadan (7°44′N, 3°83′E) and rainforest by Odeda in Ogun State (7°23′N, 3°53′E) and Bowen in Osun State (7°38′N, 4°11′E). Moreover, Abeokuta enjoys the maritime effect of the Atlantic Ocean more than Ibadan. Iwo is in the northern part of the two other cities, so it is less affected by maritime winds. The difference in the three environments also resides in the chemical composition and the types of soils.

The test environments were further assessed using the GGE model to define the relationship among the components of mega-environments, to better discriminate the best and most stable genotypes important in a breeding program. Two PCAs were involved in generating the biplot, and the maximum variation was recorded with PCA1 for all the agronomic characters. Across the locations, YPP, FPW, HSW, TNPP, LOS, and WOS are largely influenced by the genotype-by-environment effect. The distance among each tested environment is displayed in **Figure 7**, Patterns A, B, C, D, E, and F also for all evaluated traits. Similar results were obtained by Oladosu et al. (2017) and Khan et al. (2021), who reported that the GGE biplot vividly explained the existing relationships among tested locations in a genotype-by-environment analysis.

## 5 Conclusion

The 15 Bambara groundnut accessions used in the current investigation showed different variations in their responses to the three test environments due to the effects of GEI and different expressions of genes that regulate the agronomic traits. G8 was the top most-yielding genotype among the 15 genotypes. G8, G4, G7, and G13 were high yielding and the most stable across the environments. G11, G14, and G9 were unstable, but they have above-average yields. G14, G12, G15, and G1 were unstable and had below-average yields. Bowen was discriminating and representative and is classified as the superior environment. This study identified the genotypes that adapted well and uniquely to each environment. TVSU 455 (G8) and TVSU 458 (G3) recorded the highest yields in Bowen. TVSU 455 (G8), TVSU 939 (G6), and TVSU 454 (G1) were the best in Ibadan, and TVSU 158 (G2) and TVSU 2096 (G10) outperformed in Odeda. TVSU 455 (G8) was the stable variety across the three environments. They could also be used as parental lines in breeding programs.

## Data availability statement

The original contributions presented in the study are included in the article/supplementary material. Further inquiries can be directed to the corresponding author.

## Author contributions

VE designed the experiment. GO carried out the trials and collected the data. VE, GO, TO, and IO performed the analysis and wrote the manuscript. VE, TO, and IO supervised the study and reviewed the manuscript. All authors contributed to the article and approved the submitted version.
